# Replacing Corn and Wheat in Layer Diets with Hulless Oats Shows Effects on Sensory Properties and Yolk Quality of Eggs

**DOI:** 10.3389/fnut.2017.00037

**Published:** 2017-07-31

**Authors:** Louisa R. Winkler, Aimee Hasenbeck, Kevin M. Murphy, James C. Hermes

**Affiliations:** ^1^Sustainable Seed Systems Laboratory, Department of Crop and Soil Sciences, Washington State University, Pullman, WA, United States; ^2^Food Innovation Center, Oregon State University, Portland, OR, United States; ^3^Department of Animal and Rangeland Sciences, Oregon State University, Corvallis, OR, United States

**Keywords:** hulless oats, poultry diets, egg sensory properties, yolk proportion, cooked egg texture

## Abstract

US organic poultry producers are under pressure to find feed alternatives to corn and wheat. Hulless oats offer advantages such as wide geographic adaptation of the plant and high concentrations of protein and oil in the grain. They have shown considerable potential in experimental work as a feed grain for poultry, but more research is needed into their influence on the sensory and nutritional properties of eggs. In this study, hulless oats were substituted for corn or wheat at 200 g kg^−1^ in diets fed to Hy-Line Brown hens and eggs were sampled for sensory evaluation after 8 weeks. Discrimination tests of blended and baked egg samples found evidence of difference between eggs from oat-based diets and those from the oat-free control (*p* < 0.05 for eggs from an oat-corn diet, *p* < 0.01 for eggs from an oat-wheat diet). Acceptance tests of similar samples showed that eggs from the oat-wheat diet were significantly less liked than control eggs for their texture (*p* < 0.01) and response to cooking (*p* < 0.01), while eggs from the oat-corn diet were somewhat less liked. Yolk weight was greater (*p* < 0.05) in control eggs (34.1 g) than eggs from oat-corn (31.6 g) or oat-wheat (31.2 g) diets, leading to smaller yolk proportion in the oat-fed eggs. Fatty acid profile differences across treatments were not of nutritional significance, and no evidence was found that the feeding of hulless oats improved storage properties of eggs. In this study, modifying the carbohydrate source in layer diets was shown to change textural properties of cooked eggs in a way that was perceptible to untrained consumers, probably by reducing the yolk proportion. This finding was not commercially relevant owing to small effect size, and results overall add to existing evidence that hulless oats can be fed to poultry at a moderate proportion of the diet with no negative effect on consumer acceptability of eggs. Regardless of the small effect size, however, findings are interesting from the food chemistry perspective because they provide novel evidence of how the thermal properties of eggs can be altered by a change in hen dietary carbohydrate source.

## Introduction

Hulless oats (a variant of cultivated oats, *Avena sativa* L.) are of interest to poultry producers for their potential to replace corn and wheat in feed, particularly in value-added markets such as those for organic and genetically modified (GM) free products. Non-GM corn can be difficult to source in the US, since GM varieties represent over 90% of domestic corn plantings (1). No GM varieties of oats exist, making it straightforward to certify oats as a GM-free ingredient. They can be commercially produced not only within the US Corn Belt but are also adapted to cool, wet climates (2) and could, therefore, become a locally grown feed in regions where corn production is not possible and wheat production is a challenge. An additional advantage of expanding hulless oat cultivation to supply the poultry industry would be an increase in agricultural biodiversity, which has declined in the US during recent decades (3).

Hulless (naked) oats have higher concentration of protein and oil than other feed grains, including hulled (husked) oats, and have shown considerable potential in experimental work as a feed grain for poultry (4–8) and pigs (9–11). For egg producers, a primary concern is to ensure that incorporation of hulless oats in layer diets will not give rise to changes in egg sensory properties that negatively impact consumer acceptance. Secondarily, producers may also be interested in whether hulless oat inclusion leads to positive changes in egg sensory or nutritional properties which could offer marketing opportunities beyond appealing to consumer concern for environmental performance of cropping systems. To address these questions, sensory and nutritional evaluations of oat-fed eggs are required.

Reports of two such evaluations are available in the literature and both suggest that hulless oats fed at up to half of the diet by weight show no influence of commercial relevance on egg sensory properties except for a loss of yolk color intensity if hulless oats are substituted for corn (4, 12). Macleod (4) substituted wheat for hulless oats at 250 and 500 g kg^−1^ of the diet of caged Lohmann Brown and Isabrown hens; diets were equalized for metabolizable energy, lysine, methionine, arginine, tryptophan, and threonine. A 10-member panel performed triangle tests of eggs from each diet treatment against the control, and were unable to discriminate in either case. Cave et al. (12) substituted hulless oats for corn and soybean meals at levels of 0–800 g kg^−1^, equalizing the metabolizable energy, lysine, and methionine content of diets, using caged White Leghorn hens. Boiled eggs were evaluated from hens at 8 and 15 months of age by a trained sensory panel, which determined that oat inclusion did not influence sulfur aroma, mouth coating, and sour taste but was associated with lower yolk flavor than in the control (*p* < 0.05) in eggs from hens fed with the 800 g kg^−1^ diet (6.9 vs. 7.8 on a 15 cm scale) and the 600 g kg^−1^ diet (7.1 vs. 7.8), and sampled at 8 months. Yolk flavor was not rated significantly different from the control in eggs from hens fed 300 g kg^−1^ at either sampling date.

Both existing studies used small taste panels (*n* ≤ 10), one of which was a trained panel; but larger, untrained panels are thought to offer a more direct indication of market response (13). Also, in previous studies, samples were presented as boiled eggs. Recent data suggest that US consumers are as likely or more likely to prepare eggs broken out of the shell (e.g., by scrambling or frying) than by boiling in the shell (14). In this study, we revisit the hypothesis that sensory differences between eggs are not perceptible when hulless oats are used to replace corn or wheat in layer diets, and explore whether it holds under the conditions of a more rigorous test using a broken-out sample preparation method and a larger, untrained consumer panel.

Concentrations of crude protein, lipid, and macro minerals in the hen’s egg are generally not influenced by moderate changes in its diet (15), likewise the amino acid profile (16). Fatty acid profile of the egg yolk, however, is known to be influenced by that of the hen’s diet. Poureslami et al. (17) showed that yolk concentration is a direct reflection of dietary concentration in the case of n-3 fatty acids in particular, but also in the case of saturated and monounsaturated fatty acids; while yolk concentration of C18:2n-6 and monounsaturated fatty acids appear to be inversely related. Lipids are volatile precursors, and different fatty acids give rise to different volatile compounds, thereby potentially affecting egg flavor and aroma (18). Previous research addressing the influence of substituting hulless oats for corn and soy in layer diets has identified changes in yolk fat composition, namely decreased concentration of sphingomyelin in eggs from all oat-based diets [*p* < 0.05 (12)]. In the same study, carotenoid concentration of yolks from hulless oat diets was found to decrease owing to the lack of xanthophylls in oat grain. Certain carotenoids are also precursors to important flavor compounds (18), but none of the volatiles identified in eggs by Macleod and Cave (19, 20) appears to be among them. These findings suggest that should sensory differences between oat-fed and oat-free eggs be detected, fatty acids are more likely to be implicated than carotenoids.

Replacing saturated fatty acids (SFAs) in human diets with polyunsaturated fatty acids (PUFA), while maintaining appropriate proportions of n-3 and n-6 PUFA, is known to be of benefit for cardiovascular health (21). In most western populations, under-consumption of n-3 PUFA, particularly the essential alpha-linolenic acid (ALA), is the biggest challenge to optimization of dietary fat composition because food sources other than oily fish are difficult to find (21). With respect to the nutritional value of the egg, it is therefore of interest to determine whether the feeding of hulless oats to layers is associated with change in egg yolk concentration of ALA, the n-3:n-6 ratio, or concentration of SFA.

Antioxidants in poultry diets may be transferred to the egg yolk and protect against oxidative degradation of unsaturated fatty acids in the yolk and its associated off-flavors and changes in taste and texture (22). They can also increase the albumen viscosity, an indicator of albumen quality which is measured by taking the height of the albumen and converted to a Haugh Unit value that takes account of the size of the egg (23). Hulless oats included in layer diets at 250 and 500 g kg^−1^ were reported in a previous study to increase Haugh Unit values of fresh eggs and reduce rate of decline in storage relative to a wheat-based control (4). The same study found elevated levels of thiobarbituric acid reactive substances in oat-fed eggs, increasing with increasing dietary oat concentration. These findings suggest that the inclusion of hulless oats in layer diets could offer a commercially relevant advantage by improving the storage properties of eggs.

With a view to better characterizing the egg quality implications of using hulless oats as a poultry feed grain, this study evaluates (i) consumers’ ability to discriminate oat-fed from oat-free eggs; (ii) consumers’ ability to discriminate stored vs. fresh eggs from oat-based and oat-free diets; and (iii) consumers’ acceptance of oat-fed and oat-free eggs that are broken out before cooking. Yolk fatty acid profile is measured as a key component of egg nutritional value and as the parameter hypothesized to best explain potential changes in egg sensory properties associated with inclusion of oats in the hen diet. The effects of substituting hulless oats for either corn or wheat are separately evaluated, in the context of a soy-based diet typical of current commercial practice in the US poultry industry.

## Materials and Methods

### Oat Composition and Feed Analysis

Established procedures were used to analyze crude protein (combustion, AOAC 992.15 and 990.03; AOCS Ba 4e-93), crude lipids (Soxhlet extraction, AOAC 948.22), crude fiber (AOAC 962.09), and ash (AOAC 942.05) in oat, corn, and wheat grain samples. Calculated nutrient concentrations of mixed feed were based on analyzed values for oat and reference values for other ingredients (24).

### Experimental Diets and Egg Production

Three diet formulations were studied, two of which included hulless oat grain at 200 g kg^−1^ and one of which was an oat-free control resembling a typical commercial organic feed. The oat-based diets were (i) Oat + corn and (ii) Oat + wheat. This approach was chosen to distinguish whether observed results were explained by the omission of corn/wheat or the inclusion of oats. Oats were grown at Washington State University’s Northwest Washington and Extension Center at Mount Vernon, WA, USA in 2015. Other ingredients were sourced commercially. All diets contained soybean meal; a premix containing vitamins, minerals, methionine, and salt (Poultry Nutri-Balancer, The Fertrell Company, Bainbridge, PA, USA); and limestone as the calcium source. They were formulated to standardize percent content of crude protein, crude lipid, calcium, phosphorous, and energy value and to be sufficient in the sulfuric amino acids cysteine and methionine. Since the oats were high in lipids relative to other feed ingredients, soybean oil was used to supplement the lipid content of the oat-free control diet. Feeds were mixed at Oregon State University and fed in mash form.

The feeding trial in which eggs were produced took place between April and June 2016 and its methods are described in detail elsewhere (25). In brief, experimental diets were fed to Hy-Line Brown birds (a laying strain commonly used in commercial production) housed in individual cages, where a group of 10 consecutive cages with a shared feeding trough constituted a single replicate and there were three replicates per diet. Groups were arranged in a completely randomized design. Experimental diets were introduced when the hens were 24 weeks of age and were fed for 9 weeks, during which time feed and water were provided *ad libitum*.

Eggs were collected for evaluation of fatty acids from all replicates 8 weeks after the start of the experiment. These eggs were stored for 5 days at 3°C until testing. All fresh eggs for sensory evaluations were collected 2–4 days before the evaluation and stored at 3°C. Stored eggs for Discrimination 1 (“stored vs. fresh”) were collected 30 days before the evaluation and stored at 3°C. An equal number of eggs was collected from each replicate and pooled for subsampling in sensory and nutritional tests. Subsampling was random but restricted to eggs of average size and with single yolks.

All animal care procedures were approved by Oregon State University’s Institutional Animal Care and Use Committee.

### Sensory Evaluations

#### Recruitment and Test Environment

The sensory evaluation protocol received ethical approval from Oregon State University’s Institutional Review Board. Participants in consumer evaluations were recruited through Oregon State University’s sensory lab database of volunteers from the surrounding community. Volunteers were screened to ensure that they ate eggs regularly (at least 2–3 times a month), had no egg allergies, and were the primary shopper in their household. No other screening criteria were imposed, including age, because the sensory panel was intended to represent typical consumers in the study region and thereby to provide a direct reflection of market response. Compensation was offered as an incentive for participation in the study. All taste tests were carried out in a laboratory setting with each panelist in an individual booth under red lighting to mask color differences between samples. Spring water at ambient temperature was provided for panelists to cleanse their palate between samples.

#### Sample Preparation

Samples were presented as patties from bulk-blended eggs so as to avoid any individual hen effect on egg sensory properties. Four eggs per treatment were whisked together for 60 s and one-eighth of a cup was subsampled into a muffin tin in a 12-hole tray where each tray was greased with a thin layer of canola oil spray from a metered can. Eggs were baked at 180°C for exactly 12 min, then patties were transferred to ceramic plates and covered with aluminum foil until serving. No seasoning was added. For the acceptance test, preparation method was modified by (i) omitting the greasing step and (ii) ensuring that each panelist received samples taken from the same location in their separate muffin tins, so as to minimize possible effects arising from samples’ position in the oven. In all tests, egg patties were freshly prepared a maximum of 10 min prior to tasting.

This cooking method was selected to approximate typical in-home egg preparation styles such as scrambling or frying, while achieving maximum possible standardization of samples.

#### Discrimination 1: Oat vs. No-Oat

Triangle tests were carried out to evaluate the hypothesis that the substitution of hulless oats for (i) corn and (ii) wheat in layer diets causes no perceptible difference in egg sensory qualities, i.e., that consumers would be unable to discriminate between eggs from oat-containing and oat-free diets. In accordance with the triangle test methodology, the panelist was simultaneously presented with three samples, two identical and one unique, each labeled with a separate three-digit code. He or she was asked to taste the samples in a specified order and identify the unique sample from the two identical ones. Discrimination 1 involved two comparisons: (i) eggs from the Oat + corn diet vs. the Control diet; (ii) eggs from the Oat + wheat diet vs. the Control. Each panelist, therefore, tasted six samples. Panelists were also asked to note how confident they felt about their selection (multiple choice, “sure” or “unsure”) and invited to offer comments in their own words about the samples. The order of sample presentation was balanced to control for first order and carryover effects, and each sample was presented an equal number of times. Forty-five volunteers took part in this experiment: 10 males and 35 females representing age categories from 18–24 to >65 with average age range of 30–39 years. The experiment was conducted in 30-min sessions.

#### Discrimination 2: Stored vs. Fresh Eggs

Triangle tests were carried out to test the hypothesis that the substitution of hulless oats for (i) corn and (ii) wheat in layer diets improves the storage properties of eggs and, therefore, that fresh and stored eggs from oat-containing diets are indistinguishable, whereas fresh and stored eggs from the oat-free Control diet are distinguishable to untrained panelists. Stored eggs were stored for 30 days, which is close to the maximum storage duration of 5 weeks deemed safe by the US government’s Food Safety Inspection Service. The method of the triangle test was as described above, including the balancing procedure, but this experiment involved three comparisons: fresh vs. stored eggs from (i) the Oat + corn diet; (ii) the Oat + wheat diet; and (iii) the Control diet. Each panelist, therefore, tasted nine samples. Panelists were also asked to note how confident they felt about their selection (multiple choice, “sure” or “unsure”) and invited to offer comments in their own words about the samples. Forty-seven volunteers took part in this experiment: 12 males and 35 females representing age categories from 18–24 to >65 with average age range of 30–39 years. The experiment was conducted in 30-min sessions.

#### Acceptance Test

In the tasting component of the acceptance test, panelists were presented with three individual egg samples one at a time: (i) Oat + corn; (ii) Oat + wheat, and (iii) Control. For each sample, they were asked to evaluate five separate criteria. Overall liking, overall flavor and overall texture were evaluated on hedonic liking scales ranging from 1 (dislike extremely) to 9 (like extremely). Five-point Just About Right sales were used to evaluate flavor strength (1 = much too weak, 3 = just about right, 5 = much too strong) and how well cooked each sample was (1 = not nearly cooked enough, 3 = just about right, 5 = much too cooked). Panelists were also invited to give comments describing in their own words what they liked and disliked about each sample. The order of sample presentation was balanced to control for first order and carryover effects.

To test the independence of panelists’ taste evaluations from their visual impressions of samples, we included a visual component in the acceptance test. Following taste tests, panelists were taken to a separate room with natural lighting where they were shown a single egg sample from each treatment and asked to evaluate appearance on a 1–9 hedonic liking scale and color on a 1–5 Just About Right scale (1 = much too light, 3 = just about right, 5 = much too dark). Seventy-four volunteers took part in this experiment: 25 males, 48 females, and 1 transgender male representing age categories from 18–24 to >65 with average age range of 40–49 years. The experiment was conducted in 20-min sessions.

### Yolk Quality

Eggs were broken out and separated and the fresh weight of the yolk recorded. Total lipids were extracted from the egg yolk according to the method of Folch et al. (26). One gram of yolk fat was combined in a test tube with 9 mL of chloroform–methanol mixture (2:1 by volume), vortexed until homogenized, and refrigerated overnight at 4–6°C. To each sample, 2.25 mL of salt solution (0.88% NaCl) was added, and this mixture was then centrifuged at 2,000 RPM for 5 min to induce phase separation. The upper phase (water soluble fraction) was siphoned off, the plug of lipoproteins and other solids bypassed and the sample collected from the lower phase (fat-soluble liquid fraction). For conversion of total lipids to fatty acid methyl esters, 1 mL of each sample was dried under nitrogen gas and then placed in a hot water bath with 2 mL of a mixture of boron-trifluoride, hexane, and methanol (35:20:45 by volume) for 1 h. Samples were cooled to room temperature and shaken with 2 mL of HPLC grade hexane and 2 mL distilled water.

Fifty microliters taken from the top portion of the methylated sample were added to 600 µL hexane and injected into a gas chromatography machine (Agilent HP6890 gas chromatograph, Agilent Technologies Inc., Wilmington, DE, USA) with fused silica capillary column (Supelco Analystical, Bellefonte, PA, USA) for separation and quantification of fatty acid methyl esters. Equipment and methodology of the gas chromatography procedure are described by Cherian and Sim (27). Fatty acid methyl esters were identified by comparison with retention times of authentic standards (Nucheck Prep, Elysian, MN, USA) and the data processed with HP ChemStation software (Agilent Technologies Inc., Wilmington, DE, USA). Individual fatty acids values are expressed as percentages of total fatty acid methyl esters in each sample.

### Statistical Analysis

All statistical tests were implemented in the R statistical software environment (28).

#### Sensory Evaluations

Discrimination test data were evaluated for significance against the null hypothesis that the samples were the same (probability of true discriminators = 0) using an exact binomial test. Power of the discrimination test was calculated based on the *p*-value obtained. Tests were implemented in package “sensR” (29). Narrative comments were examined for key words and themes.

For the acceptance test, association between panelists’ ratings and hen diets was tested according to a repeated measures design because each panelist rated three eggs. Using the R package “nlme” (30), a mixed linear model was constructed with diet as a fixed effect and panelist as random and compared to a baseline model without the diet term to evaluate overall significance of diet. For attributes showing a significant effect of diet, *post hoc* means separation was carried out using Tukey’s test of Honest Significant Difference, implemented in package “multcomp” (31). Least-squares estimates for the rated attributes were extracted from models and used to evaluate correlations between them, carried out using the “psych” package (32). All model residuals were visually inspected to ensure that model assumptions were met.

#### Yolk Weight and Fatty Acids

Results were subjected to one-way analysis of variance using diet as the sole fixed effect in the model. Fisher’s least significant difference was used for *post hoc* means separation.

## Results

### Experimental Diet Composition

Although efforts were made to formulate experimental diets for equal metabolizable energy and protein content, calculated nutrient concentrations displayed in Table 1 suggest that Oat + wheat diets may have been deficient in protein, particularly in the essential amino acid lysine. In other respects, experimental diets were appropriate to the physiological needs of laying hens.

**Table 1:** Experimental diet ingredients and calculated nutrient composition.

	Control	Oat + corn	Oat + wheat	

	**Ingredients (% by weight)**			
Oats	–	20.0	20.0	
Corn grain (yellow)	45.0	48.2	–	
Wheat grain (soft white)	23.3	–	52.3	
Soy meal	19.3	20.0	13.3	
Vitamin and mineral premix	3.0	3.0	3.0	
Limestone	7.5	7.5	7.5	
Soybean oil	2.0	1.3	4.0	

**Nutrient concentration, calculated values (%)**				**Requirement^a^**

M.E. (kcal g^−1^)	2.88	2.87	3.00	2.80
Protein (%)	14.74	15.48	14.45	15.04
C18:2n-6, linoleic acid (%)	2.22	2.34	2.92	0.88
Ca (%)	3.36	3.37	3.37	3.72
Total P (%)	0.59	0.58	0.59	–
Available P (%)	0.37	0.38	0.38	0.41
Ca:P	8.98	8.92	8.92	9.07
Cl (%)	0.22	0.23	0.24	0.16
Na (%)	0.15	0.15	0.17	0.16
Lysine (%)	0.70	0.77	0.63	0.80
Methionine (%)	0.34	0.36	0.30	0.39
Methionine + cysteine (%)	0.59	0.66	0.58	0.71
Threonine (%)	0.46	0.57	0.51	0.61
Arginine (%)	0.85	1.05	0.94	0.82
Isoleucine (%)	0.65	0.74	0.69	0.62
Valine (%)	0.71	0.82	0.76	0.71

*^a^Requirements are taken from the Hy-Line Management Guide for Hy-Line Browns in peak production feeding phase, based on hen.day consumption of 113 g (the maximum available value, but less than was consumed in this study)*.

### Sensory Evaluation

#### Discrimination 1: Oat vs. No-Oat

In a triangle test, the null hypothesis is that all panelists choose randomly between the three options presented to them and their choices are correct one-third of the time. Close to one-half of all panelists correctly differentiated Oat + corn eggs and Oat + wheat eggs from the oat-free Control in the Oat vs. No-oat discrimination experiment (Table 2). In both cases, the number of correct selections was significantly greater than the null expectation of one-third correct choices. The proportion of panelists choosing correctly who were confident of their choice was 55% in the case of Oat + corn eggs and 48% in the case of Oat + wheat eggs, suggesting that Oat + wheat eggs may have been more distinguishable from the Control than Oat + corn but at the same time that the difference was not clear in either case. The parameter *d*′ is a standardized representation of underlying sensory difference between samples calculated from the data according to a Thurstonian modeling approach, and has a maximum value of 5.0 (33). In this study, *d*′ was estimated at 1.41 for Oat + corn vs. Control and 1.52 for Oat + wheat vs. Control (Table 2).

**Table 2:** Discrimination test results for comparisons of eggs from (i) Oat + corn and (ii) Oat + wheat diets to eggs from the oat-free Control diet.

Treatment group	*n*	Correct responses^a^	% “Sure,” correct responses^b^	% “Sure,” incorrect responses^b^	*d*′ (95% CI)^c^
Oat + corn	45	22*	55	43	1.41 (0.20–2.19)
Oat + wheat	45	23**	48	41	1.52 (1.44–1.60)

^a^Statistical significance of result at *p* < 0.05 (*), *p* < 0.01 (**).

^b^Percent of respondents stating they felt sure of their selection in response to a multiple choice question with options “sure” and “unsure.”

*^c^*d*′ is the Thurstonian standardized estimate of sensory difference between samples on a 0–5 scale, presented here with its 95% confidence interval (CI)*.

Examination and interpretation of panelists’ narrative comments revealed several recurring themes (Table 3). Panelists were more likely to comment that the oat-fed egg had more flavor than the control, though the difference was greater for Oat + corn than Oat + wheat. Comments were more likely to characterize the control sample as having a fluffier, less rubbery texture, and to characterize the oat-fed samples as being more browned, cooked and/or crispy.

**Table 3:** Incidence of selected themes^a^ appearing in open-ended comments of panelists who correctly discriminated oat-fed eggs from oat-free control eggs in Discrimination 1.

	Oat + corn vs. Control (*n* = 23)^b^	Oat + wheat vs. Control (*n* = 22)^b^
	
	% of Comments	
Control had more flavor (including more “eggy”)	9	14
Oat-fed had more flavor (including more “eggy”)	35	27
Control had fluffier texture (including less “rubbery”)	13	27
Oat-fed had fluffier texture (including less “rubbery”)	4	0
Control seemed more browned/cooked/crispy	4	0
Oat-fed seemed more browned/cooked/crispy	26	14

*^a^Themes were selected as the most commonly occurring based on examination and interpretation of panelists’ comments*.

*^b^n is the number of correct responses*.

#### Discrimination 2: Stored vs. Fresh Eggs

Results indicated that panelists were not able to distinguish fresh from stored eggs in any of the three treatment groups (Table 4). More panelists correctly identified the odd sample in the Oat + corn test (*n* = 20) than in Oat + wheat and Control group tests (both *n* = 15), but the difference from the null expectation of one-third correct choices (*n* = 16) was not statistically significant. Also, the proportion of panelists feeling sure of their selection in the Oat + corn test was lower among correct than incorrect panelists (40 vs. 45%).

**Table 4:** Discrimination test results for comparisons of 30-day stored to fresh eggs from (i) Oat + corn, (ii) Oat + wheat, and (iii) oat-free Control diets.

Treatment group	*n*	Correct responses^a^	% “Sure,” correct responses^b^	% “Sure,” incorrect responses^b^
Oat + corn	47	20 NS	40	45
Oat + wheat	47	15 NS	53	47
Control	47	15 NS	53	50

*^a^NS indicates that result was not statistically significant at alpha level of 0.05*.

^b^Indicates % of respondents stating they felt sure of their selection in response to a multiple choice question with options “sure” and “unsure.”

#### Acceptance Test

**Figure 1:**
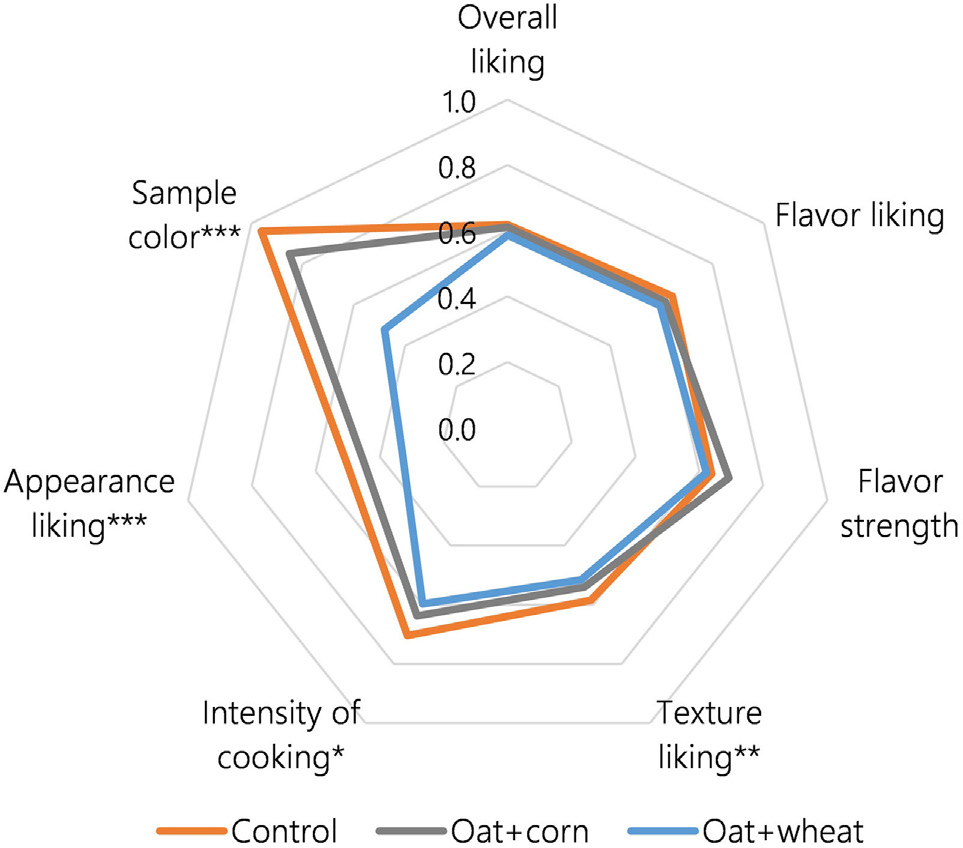
Visualization of acceptance test results (*n* = 74 panelists) for two oat-based diets and an oat-free control. The greater the area within the lines, the higher the consumer desirability of the entry. The figure was prepared by converting all scores to a 0–1 scale: Liking scores (Appearance, Overall liking, Flavor liking, Texture liking) were scaled by dividing the absolute score (1–9) by 10; JAR scores (Color, Flavor strength, Degree of cooking) were scaled by taking the absolute value of the difference between the score (1–5) and the “ideal” value (3) and dividing by 10. Where scores of different diets were found to differ significantly from one another according to the Tukey HSD test, * indicates *p* < 0.05; ** indicates *p* < 0.01; *** indicates *p* < 0.001.

Acceptance test results are visualized in Figure 1; full results and test questions are reported in Table S2 in Supplementary Material. Overall liking (Q1), flavor liking (Q2), and flavor strength (Q3) scores were not significantly different across treatment groups. Liking score for texture (Q4) was significantly higher for Control than Oat + wheat samples (*p* < 0.01, 5.85 vs. 5.16). For intensity of cooking, a similar pattern was observed, with Control sample scores falling significantly closer to “just about right” than Oat + wheat sample scores (*p* < 0.05, 3.42 vs. 3.68). Panelists scored all samples as more than adequately cooked, i.e., tending toward overcooked.

**Figure 2:**
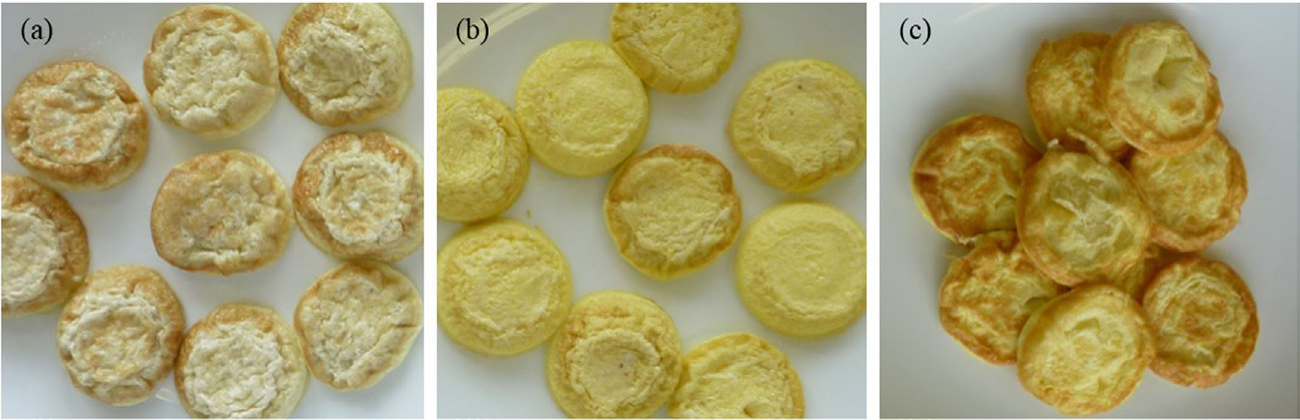
Samples prepared by the method described in this study; eggs from **(A)** Oat + wheat, **(B)** Oat + corn, and **(C)** Control diets. Photographs: Louisa Winkler.

Liking scores were low in general, ranging between “dislike moderately” and “like slightly.” In particular, panelists were not attracted to the appearance of samples (Figure 2). The lowest appearance liking scores (Q6) were given for Oat + wheat eggs, which had the least intensity of yolk color, and highest for Control eggs (*p* < 0.001; 3.30 vs. 5.01). Panelists’ direct response to sample color (Q7) followed a similar trend, with both Control (3.04) and Oat + corn (3.18) samples scoring significantly closer to “just about right” than Oat + wheat samples (1.92, *p* < 0.001).

There was no correlation between color JAR scores and overall liking (Table S2 in Supplementary Material), suggesting that the experimental protocol successfully prevented panelists’ perceptions of yolk color from influencing their ratings of flavor and texture. At the same time, appearance liking scores showed significant correlations (*p* < 0.001) with both overall liking (*R* = 0.29) and flavor liking (*R* = 0.30).

Of the characteristics tested, flavor liking had the strongest correlation (*R* = 0.83, *p* < 0.001; Table S2 in Supplementary Material) with panelists’ overall liking of samples. Texture also appears to have been influential (*R* = 0.64, *p* < 0.001). This finding was reflected in panelists’ narrative responses to questions about each sample (Table 5). Panelists showed a stronger tendency to report hard or rubbery texture—a negative characteristic—for Oat + wheat samples than for Oat + corn or Control samples. Positive general comments on cooking characteristics occurred most frequently for Control samples and least frequently for Oat + wheat samples. With regard to flavor, panelists made the most positive and the least negative comments about the Oat + wheat samples, though there was a small tendency for the control to be ascribed more “yolk” flavor.

**Table 5:** Incidence of selected themes^a^ appearing in open-ended comments of 74 panelists in an acceptance test of cooked eggs from three diets with and without hulless oats, expressed as% of comments in which theme appears.

	Diet		
	Control	Oat + corn	Oat + wheat
**In response to “what do you dislike about this sample?”**			
General cooking result^b^	39	46	38
Hard or rubbery	14	24	46
Not enough flavor, bland	31	34	28
Does not have “yolk” flavor	1	1	3
**In response to “what do you like about this sample?”**			
General cooking result	19	14	7
Appealing flavor	16	19	22
Has “yolk” flavor	3	1	1

*^a^Themes were selected as the most commonly occurring based on examination and interpretation of panelists’ comments*.

*^b^Characteristics mentioned included: deflated, difficult to cut with fork, overcooked, artificial texture, too chewy, undercooked, slimy*.

### Yolk Quality

Yolk quality data are displayed in Table 6. The threshold adopted for declaring significant difference between treatment group means was *p* < 0.05. Yolk weight of control eggs was greater than that of either oat-based diet. Major fatty acids were oleic (42.70% on average of yolk fatty acids), followed by palmitic (23.87%) and linoleic (16.21%) acids. Linoleic acid was significantly higher in Oat + wheat eggs than in the Control and tended also to be higher in Oat + corn eggs. Total n-6 fatty acids were significantly enriched in Oat + wheat eggs relative to the Control and tended also to be enriched in Oat + corn eggs. However, this difference did not result in significantly different n-6/n-3 ratios across treatment groups. Docosapentaenoic acid (C22:5n-3) was significantly higher in Oat + corn eggs than the Control and tended to be higher in Oat + wheat eggs. Eicosapentanoic acid (C20:5n-3) was present at very low levels in the samples (maximum value 0.35% of total fatty acids) and was not reliably detected by our equipment, leading to missing data; this fatty acid is, therefore, not reported.

**Table 6:** Yolk quality characteristics of raw eggs from hens fed one of three diets.^a^

	Control	Oat + corn	Oat + wheat	SD^b^
Yolk weight (g)	34.1_a_	31.6_b_	31.2_b_	1.64

	**Fatty acids (% of total)^c^**			

C14:0	0.28	0.29	0.29	0.02
C16:0	24.52	23.73	23.37	1.21
C16:1	3.71	3.43	3.38	0.25
C18:0	8.73	9.00	9.17	0.46
C18:1	43.04	42.90	42.17	0.82
C18:2n-6	15.26_b_	16.21_ab_	17.15_a_	0.99
C18:3n-3	0.86	0.61	0.72	0.21
C20:4n-6	2.01	2.09	2.04	0.10
C22:5n-3	0.26_b_	0.42_a_	0.29_b_	0.08
C22:6n-3	1.25	1.21	1.34	0.08
Total n-3	2.37	2.25	2.35	0.20
Total n-6	17.26_b_	18.31_ab_	19.19_a_	1.02
n-6/n-3	7.37	8.16	8.17	0.66
Total PUFA	19.63	20.55	21.54	1.07

*^a^Entries bearing different subscript letters within a row are significantly different according to Fisher’s test of least significant difference (*p* < 0.05)*.

*^b^SD is the pooled standard deviation*.

*^c^C14:0 = myristic acid; C16:0 = palmitic acid; C16:1 = palmitoleic acid; C18:0 = stearic acid; C18:1 = oleic acid; C18:2n-6 = linoleic acid; C18:3n-3 = alpha-linolenic acid; C20:4n-6 = arachidonic acid; C22:5n-3 = docosapentaenoic acid; C22:6n-3 = docosahexaenoic acid; total n-3 = C18:3n-3 + C22:5n-3 + C22:6n-3; total n-6 = C18:2 + C20:4n-6; PUFA = polyunsaturated fatty acid*.

## Discussion

Sensory differences observed between treatment groups in this study were not great. Overall and flavor liking in the acceptance test did not differ significantly between treatments, and Oat + corn eggs did not differ from the Control in any of the characteristics evaluated. In the discrimination test, there was a high level of uncertainty even among panelists who correctly identified the odd sample.

Nonetheless, results from both the discrimination and acceptance tests indicated that while small, certain differences between eggs from the three experimental diets were statistically detectable in the laboratory environment of the sensory analysis. Based on scores from the acceptance test and comments from both discrimination and acceptance tests, these differences were primarily related to texture and the intensity of cooking, with Oat + corn eggs and especially Oat + wheat eggs having a greater tendency to be harder and more “rubbery.” This finding suggests that chemical differences between eggs from different diets could have influenced their thermal properties and, hence, their response to the cooking method used in this study.

A factor likely contributing to different thermal and sensory properties of treatment groups was yolk proportion. Enrichment in linoleic acid is reported to increase egg size by enlarging the albumen, thereby decreasing yolk proportion (15). Such a relationship was observed in this study, where inclusion of hulless oats was associated with 0.09-fold average enrichment in linoleic acid and significantly lighter yolk weight (*p* < 0.05, Table 6). Average shell-on egg weights in oat-fed diets were reported to be higher than that of the control, while shell thickness did not differ between groups, strongly suggesting reduced yolk proportion in oat-fed eggs (25). The albumen is probably the major source of browning reactions in a cooked whole-egg mix, since it contains around half of the egg’s proteins and 99% of its carbohydrates, molecules that together participate in browning reactions (34). This may explain why panelists tended to describe oat-fed eggs, with their higher albumen proportion, as more browned/cooked/crispy than the control (Table 3), and perhaps also why they tended to prefer the texture and cooking intensity of the control (Figure 1; Table S2 in Supplementary Material). Feeding hulless oats together with peas (*Pisum sativum* L.), which can reduce egg size (15), might be one strategy to redress the change in yolk proportion of oat-fed hens and represents an opportunity for further research.

Variations in the molecular composition of the egg may also be implicated in thermal response. The egg yolk is primarily composed of lipids [65% dry matter (34)]. The lipid proportion of the egg yolk is largely stable, but fatty acid composition can alter in response to diet. We observed significant changes in fatty acid composition across treatment groups in this study (Table 6). Previous experimental work has shown that changes in fatty acid composition affect firmness of cooked yolks, though the mechanisms are complex and it is unclear exactly how they could be implicated in our results (35, 36). In the egg white, proteins represent the major fraction (90% dry matter) and are, therefore, the most important determinant of its thermal properties. Protein composition influences coagulation and viscoelastic properties of egg whites, since each of the 148 proteins found in egg white has its own chemical and physical properties such as denaturation temperature and isoelectric point (37). The hen’s diet appears to have only a constrained effect on total protein and protein composition of the albumen (16, 38), but it shows stronger influence on concentrations of fat- and water-soluble vitamins and certain minerals in the egg, thereby also influencing the properties of egg white during and after heating (15). Research has examined the effect on egg white functional properties of manipulating individual amino acids in hen diets. For example, Prochaska et al. (39) found that albumen proteins and solids increase, and heat-treated albumen forms harder gels, when hens are fed 1,062 mg day^−1^ lysine compared to 638 mg day^−1^, while Hammershøj et al. (37) and Shafer et al. (40) found that varying the level dietary methionine could change the albumen component yield but did not affect the functionality of egg white in cake or the texture of cooked albumen gels. To our knowledge, research into the effect on egg white thermal properties of altering major dietary components, such as carbohydrate or protein source is lacking.

Synthesizing results from Discrimination test 1 and the acceptance test, we observed greater difference from Control eggs to Oat + wheat than to Oat + corn eggs. Our results, therefore, suggest that the inclusion of oats was not the only influential treatment factor in sensory tests; but it is less clear whether the omission of corn, the addition of wheat, the unexpectedly low level of protein in the Oat + wheat diet, or some combination of these factors should be invoked. Comparison of wheat with other cereals in layer diets does not appear to alter egg physical traits, such as weight, size, or albumen height (41, 42). However, we are not aware of studies addressing cooked egg sensory properties.

Previous work has suggested that antioxidants contained in oat grains could transfer to the egg and increase albumen viscosity (measured by Haugh Units) (4) and that enrichment in antioxidants protects the yolk against oxidative degradation and development of off-flavors (22). These reports informed this study’s hypothesis that inclusion of hulless oats in layer diets could improve egg storage properties, decreasing perceptible difference between fresh and 30-day stored eggs from oat-based diets relative to controls.

However, Discrimination test 2 of stored vs. fresh eggs indicated no perceptible difference between samples. Tests of Haugh Unit values on eggs from the same feeding trial as that from which eggs for this study were obtained showed little evidence of difference across treatment groups (25). This could be because corn also contains antioxidant compounds such as zeaxanthin (43), which may have contributed to improved keeping quality in the control eggs. It, therefore, appears that while hulless oats may possess antioxidant properties, their inclusion at 200 g kg^−1^ in layer diets is not associated with significant sensory differences from a commercially typical corn-based control. Thus, their contribution may not be of marketable value to producers at this level of inclusion.

Similarly, the changes in fatty acid composition observed in eggs from diets containing 200 g kg^−1^ hulless oats were of relatively small magnitude. In a previous study, dietary manipulations gave rise to changes in egg yolk SFA and linoleic acid proportion of 1.4- and 1.6-fold, respectively, between maxima and minima (17); corresponding values in this study were 0.02 and 0.12. So far as analysis of fatty acids was concerned, we observed no negative influence of hulless oat inclusion in layer diets. Consumers are generally recommended to substitute SFA in the diet for PUFA (44), and a promising trend in our results was the increase in PUFA as a proportion of total fatty acids associated with oat-based diets, apparently explained by the displacement of palmitic, palmitoleic and oleic acids by linoleic acid (Table 6). This effect approached statistical significance (*p* = 0.06) and could potentially be intensified by increasing the proportion of hulless oat in the diet. However, for a “High Polyunsaturated Fat” health claim according to, e.g., the current European Union standard, a food item must contain 45% PUFA as a proportion of total fatty acids (45), far exceeding levels in this study of 19.63–21.85%.

## Conclusion

This study was conducted to explore the potential of adding value to eggs by incorporating hulless oats in layer diets, particularly in response to markets for GM free and environmentally friendly foods. In contrast to previous studies, eggs were prepared by breaking out and blending instead of by boiling; and sensory panel recruitment was designed to directly predict market response among typical consumers in the study region of the US Pacific Northwest (i.e., to assemble a large, age-diverse panel of untrained consumers).

Generally, we observed negligible difference between the treatments in terms of their sensory properties; decreased liking scores associated with the Oat + wheat diet should be interpreted with caution as this diet showed an unexpectedly low protein content that may have influenced egg sensory properties.

In both Oat + corn and Oat + wheat treatment groups, inclusion of hulless oat appears to have influenced thermal properties and thereby response to cooking of eggs. This finding represents novel evidence of the direct relationship between hen diet, egg thermal properties, and consumer acceptance. Our results appear to be explained by the reduced yolk proportion of oat-fed eggs, though a thorough compositional analysis would be required to develop a more complete explanation for the observation that these eggs appeared to form firmer gels and undergo more browning reactions than control eggs.

A small increase in PUFA as a proportion of total fats could be interpreted as nutritionally positive but was insufficient to earn an official health claim.

Nutritional and sensory changes reported in this study appear unlikely to represent a marketing opportunity for producers interested in using hulless oats in layer diets. Producers seeking to market their use of hulless oats will instead need to focus on their contribution to a diversified farming system and their potential to represent an option for locally grown organic and non-GM feeds.

Overall, our findings add to existing evidence that the inclusion of hulless oats in layer feeds at moderate levels shows no negative influence on the sensory properties of eggs, as long as attention is paid to the nutritional composition of diets. In this respect, hulless oats represent a viable alternative to current feed options. However, it is noted that hen diets in this study contained just 200 g kg^−1^ hulless oats and that the small changes we observed in thermal properties could become more pronounced at higher substitution levels, underlining the need for further research to avoid potential consumer acceptance issues should hulless oats be pursued as a feed grain.

## Ethics Statement

Sensory tests in this study were carried out in accordance with the recommendations of the Human Research Protection Program of Oregon State University’s Institutional Review Board with written informed consent from all subjects. All subjects gave written informed consent in accordance with the Declaration of Helsinki. The protocol was approved by the OSU Human Research Protection Program. The poultry feeding trial from which eggs were obtained for sensory tests was carried out in accordance with the recommendations of the Institutional Animal Care and Use Committee of Oregon State University’s Office of Research Integrity. The protocol was reviewed by the Institutional Animal Care and Use Committee.

## Author Contributions

LW coordinated the study and led statistical analysis and interpretation and manuscript preparation. JH and LW designed the feeding experiment and JH led its implementation. AH and LW designed the sensory experiments and AH led their implementation. KM oversaw the study and contributed to all experimental design. All authors read and approved the final manuscript.

## Conflict of Interest Statement

The authors declare that research for the submitted work was conducted without payment or services from third parties relating to any aspect herein; that they hold no financial relationships with entities that could influence or be perceived to potentially influence its content; that they hold no current or pending patents or copyrights related to this work; and that they are not engaged in other relationships or activities that influence or could be perceived to influence the submitted work.
