# Supplementary material for: Replacing Corn and Wheat in Layer Diets with Hulless Oats Shows Effects on Sensory Properties and Yolk Quality of Eggs
Source: Front Nutr. 2017 Jul 31;4:37. doi: 10.3389/fnut.2017.00037 (PMC5534467; doi:10.3389/fnut.2017.00037)
Supplement: Supplementary file 1 [file Table_1.pdf]

Table S1. Acceptance Test results (n = 74 panelists)

Question	Response scale[1]	Diet[2]			
		Control	Oat+corn	Oat+wheat	p[3]
1. Overall, how much do you like or dislike this sample? [4]	Liking	6.19	6.11	5.86	NS
2. How much do you like or dislike the overall flavor of this sample?[4]	Liking	6.42	6.16	5.96	NS
3. Flavor strength[4]	Just about right	2.43	2.55	2.39	NS
4. How much do you like or dislike the overall texture of this sample? [4]	Liking	5.85_a	5.41_{ab}	5.16_b	**
5. Intensity of cooking[4]	Just about right	3.42_a	3.57_{ab}	3.68_b	*
6. How much do you like or dislike the appearance of this sample? [5]	Liking	5.01_a	4.51_a	3.30_b	***
7. Sample color[5]	Just about right	3.04_a	3.18_a	1.92_b	***

[1]Liking scores were on a 1-9 scale, JAR scores were on a 1-5 scale. See Methods and Materials for details.

[2]Different subscript letters within a row indicate means significantly different from each other.

[3]p-value codes: NS = not statistically significant; * $p<0.05$, ** $p<0.01$, *** $p<0.001$.

[4]Evaluated in taste test.

[5]Evaluated in visual test.
