# Supplementary material for: Replacing Corn and Wheat in Layer Diets with Hulless Oats Shows Effects on Sensory Properties and Yolk Quality of Eggs
Source: Front Nutr. 2017 Jul 31;4:37. doi: 10.3389/fnut.2017.00037 (PMC5534467; doi:10.3389/fnut.2017.00037)
Supplement: Supplementary file 2 [file Table_2.pdf]

Table S2. Correlations (R) between Acceptance Test question scores[1]

	Flavor (liking)	Flavor strength (JAR)	Texture (liking)	Cooking (JAR)	Appearance (liking)	Color (JAR)
Overall (liking)	0.83***	0.30***	0.64***	-0.29***	0.29***	0.06
Flavor (liking)		0.39***	0.51***	-0.17	0.30***	-0.01
Flavor strength (JAR)			0.14	-0.02	0.15	0.04
Texture (liking)				-0.56***	0.37***	0.12
Cooking (JAR)					-0.29***	-0.09
Appearance (liking)						0.31***

******* indicates correlations are statistically significant (p<0.001).

[1]Tests were performed on least squares estimates extracted from mixed linear models.
